# Tracking Telehealth Needs for Individuals With Sickle Cell Disease Through the COVID‐19 Pandemic: A Cross‐Sectional Survey Study

**DOI:** 10.1002/hsr2.70163

**Published:** 2024-11-05

**Authors:** Marsha Treadwell, Lisa Du, Yemi Lawrence, Maxine Gaspar, Kathryn Hassell, Sanjay Shah, Moses Akpan, Nicole Crook, Marcia Taylor, Srila Gopal, For the Pacific Sickle Cell Regional Collaborative

**Affiliations:** ^1^ Department of Pediatrics University of California San Francisco Oakland California USA; ^2^ University of California San Francisco Benioff Children's Hospitals Oakland California USA; ^3^ School of Medicine University of California San Francisco San Francisco California USA; ^4^ School of Medicine University of Colorado Anschutz Medical Campus Aurora Colorado; ^5^ Sickle Cell Program, Phoenix Children's Hospital Phoenix Arizona USA; ^6^ Sickle Cell Foundation of Arizona, Inc Tucson Arizona; ^7^ Center for Inherited Blood Disorders Orange California USA; ^8^ Sickle Cell Anemia Foundation of Oregon Portland Oregon USA; ^9^ University of California San Diego Health San Diego California USA

**Keywords:** access to care, quality of care, sickle cell disease, telehealth

## Abstract

**Background and Aim:**

Pervasive disparities characterize sickle cell disease (SCD) care, including limited access to SCD specialists. Rapid deployment of remote healthcare provision and support during the COVID‐19 pandemic provides an opportunity to understand telehealth barriers/facilitators for SCD. We aimed to evaluate telehealth experiences and satisfaction for routine visits among adults and caregivers of children with SCD within a US regional collaborative.

**Methods:**

151 adults ≥ 18 [median (IQR) = 36 (28, 43) years], and 94 caregivers [median child age (IQR) = 12 (7, 14) years] completed a 30‐item online survey in 2021 assessing systems issues such as reliable Internet; health information sharing; and consumer focus (e.g., visit started on time). A multivariable logistic regression model was used to evaluate relations between independent variables and the outcome overall satisfaction with telehealth.

**Results:**

Patients were primarily diagnosed with sickle cell anemia (60.8%) and prescribed hydroxyurea (57.6%). Satisfaction with telehealth was high (> 90%), but 60.6% of caregivers gave the highest rating compared with 44.9% of adults (*p* = 0.02). Few accessibility/technical issues were cited, however, caregivers reported more often having home support for telehealth (80.9% vs. 63.6%, *p* = 0.003). In multivariable analyses, participants seen in large centers (> 200) were more likely to give the highest satisfaction ratings compared with those in smaller centers (< 100, aOR: 2.33; 95% CI: 1.21, 4.48, *p* = 0.01); however, those who needed help from a telehealth navigator were less likely to give the highest telehealth experience rating versus those who did not need help (aOR: 0.37; 95% CI: 0.19, 0.71, *p* = 0.003).

**Conclusion:**

Views of telehealth were favorable, although caregivers reported greater satisfaction and resources compared with adults with SCD. It remains important to identify factors related to telehealth utilization and efficacy for SCD populations in varied geographies and settings, to ensure equity in access.

## 1 Introduction

Ensuring that individuals with sickle cell disease (SCD) have access to specialty care has been challenging. The SCD population is predominately comprised of minoritized racial and ethnic groups in the United States, and they are often socioeconomically disadvantaged, with about 70% on public insurance [1]. Frequent complications associated with SCD include severe acute and chronic pain and progressive organ damage, resulting in increased hospitalizations and emergency department (ED) visits [2]. Disparities in access to routine primary and specialty healthcare for those with SCD has translated into increased mortality and poor quality of life, beginning as adolescents transition to adult care and continuing throughout adulthood [3].

There are few SCD specialists trained and willing to care for adults with SCD, resulting in much care being delivered in EDs and other non‐specialty settings [1, 4]. As a consequence of efforts to slow the spread of COVID‐19 and protect their health, many patients with SCD were unable to receive regular in‐person care from their specialists early in the pandemic [5]. At the same time, EDs, which have been used as care settings of last resort for patients with SCD, were overburdened with patients with COVID‐19 and also regarded as high‐risk environments [6]. In light of these limitations, many SCD specialty care visits were transitioned to telehealth (the use of electronic information and communication technologies to allow remote provision and support of healthcare) facilitated by temporary changes to insurance policies and rapid deployment of appropriate technology and training [7, 8]. The use of telehealth was initially poorly charted territory for both SCD providers and their patients, and the continuation of this approach, as the pandemic has moved into the endemic phase, remains variable. Early studies showed telehealth to be successful for SCD symptom management, to increase access to care, and to be associated with high patient satisfaction. [9, 10, 11]

These early studies were conducted with adults with SCD in middle and southern Georgia and were designed to reach medically underserved, rural communities. Similarly, a more recent study evaluated the feasibility of telehealth for children with SCD, connecting a rural “hub” site to the only SCD specialty care “spoke” clinic in the state, located in a large, urban, academic medical center over 160 miles away [12]. The 10 participating patients/families had much‐improved visit attendance (from about 50% to 100%) and they highly rated their experiences with telehealth. Shah, O'Dwyer, and S. M. Badawy conducted a systematic review of 32 studies of telehealth for pediatrics and adults with malignant and nonmalignant hematology conditions [13]. Telehealth interventions were wide‐ranging, including web‐based portals, videoconferencing, and telephone‐based interventions with outcomes including satisfaction, cost–benefit, survival, feasibility, and adherence with follow‐up. Many studies focused on patient populations that required routine monitoring due to the chronic nature of their conditions. Findings from the review underscored that affected individuals, their families, and healthcare providers—particularly, from rural communities—generally rated their experiences as favorable, and patient outcomes were better or comparable to those who did not use telehealth.

The present study examines telehealth in the Pacific Sickle Cell Regional Collaborative (PSCRC [14]), a geographically vast region funded by the Health Resources and Services Administration (HRSA) to increase access to quality care for individuals with SCD. The PSCRC is one of HRSA's five Sickle Cell Disease Treatment Demonstration Programs (SCDTDP) and includes 13 western states (Alaska, Arizona, California, Colorado, Hawaii, Idaho, Montana, Nevada, New Mexico, Oregon, Utah, Washington, Wyoming). Early in the COVID‐19 pandemic, community‐based organizations (CBOs), and community health workers (CHWs) in five PSCRC states conducted a survey study to assess the impact of telehealth on SCD care [15]. CBO leads in California, Colorado, Oregon, Nevada, and Arizona contacted clients in their database to complete a 17‐item survey by phone in May of 2020. Participants indicated if they had ever heard of or participated in telehealth; if they had access to the Internet, Wi‐Fi, and devices to access telehealth; and if they needed assistance with navigating telehealth. Participants also reported on their healthcare utilization, for example, if they had visited the ED, managed pain at home, and/or reached out to their SCD provider.

Participants in 2020 were 199 adults or parents/caregivers of children with SCD. Fifty‐five percent of those surveyed indicated that they had heard of or had participated in telehealth services; 85% reported familiarity or the ability to download Zoom; 95% reported access to Wi‐Fi and indicated they would access telehealth services through any of the following devices: mobile phones (71%), computers (59%), or tablets (17%). Survey respondents also indicated they had assistance at home with accessing telehealth sessions (64%) while the remaining (46%) indicated that they would be interested in having a CHW provide them with support in getting started with telehealth services. Over half (53%) reported they had managed SCD‐related pain at home, indicating they had reached out to their SCD providers for advice and care recommendations.

The goals of the present study were to evaluate associations between patient/family, clinical site and telehealth session characteristics, and telehealth satisfaction with routine visits among adults and parents/caregivers of children with SCD within the PSCRC. We expected that adults and parents/caregivers of children with SCD would report favorably about their care as associated with telehealth 1 year into the pandemic. Furthermore, we expected that the usability of telehealth would influence their satisfaction and access to routine care. Identifying factors related to telehealth utilization and its efficacy for the population with SCD continues to be important in progressing toward the goal of increasing access to care beyond the pandemic.

## 2 Methods

### 2.1 Participants

Recruitment for completion of the telehealth survey occurred within eight PSCRC sites in six states between March 1 and July 15, 2021. The *Impact of COVID‐19 and Telehealth* survey was designed by the SCDTDP Regional and CBO leads and consisted of multi‐option choices and open‐ended questions. The study was introduced to patients and families by clinical providers, CHWs, or clinical research coordinators (CRCs). Surveys could be completed online using a link that was emailed or texted or could be administered over the phone or in‐person by CHWs or CRCs. Central approval was obtained from the UCSF Institutional Review Board (IRB), before study startup. After participants reviewed a summary of the study's nature, potential risks and benefits, costs and compensation, and voluntariness of participation, they gave consent.

Participant inclusion criteria included: (1) individuals with a diagnosis of SCD (hemoglobin SS, Sβ^0^ thalassemia, Sβ^+^ thalassemia, SC, SD, SE, and other variants); (2) parents of minor children with an SCD diagnosis; and (3) had a telehealth visit within the period of study with their regular SCD provider. Exclusion criteria included: (1) individuals who did not have SCD or (2) individuals with SCD who were deemed by their SCD provider as unable to complete the survey for reasons such as cognitive impairment. Online surveys were in English but could be administered in‐person or over the phone in Spanish by a CHW.

### 2.2 Sites and Telehealth

PSCRC sites of care were adult (*n* = 2), pediatric (*n* = 3), or lifespan‐focused (*n* = 4); academic (*n* = 6), or community (*n* = 3); and varied by size: small (*n* = 4 with fewer than 100 patients), medium (*n* = 3 with 100–200 patients), or large (*n* = 2 with more than 200 patients). Telehealth sessions at all sites used the direct‐to‐consumer model and Zoom for Healthcare was the primary platform used, followed by Telehealth at Epic Care. Telehealth visits were defined as “medical consultation (taking place) remotely using technology,” that is, real‐time audio and visual [16]. The most common practice across all sites was to switch to phone for audio if there were issues with the video visit or switch entirely to phone if technical issues could not be solved.

Given our collaborations between clinical sites and sickle cell CBOs within the PSCRC, we defined telehealth navigators as CHWs who could assist patients/families to address barriers to telehealth participation. CHWs already assisted clients with navigating the healthcare system and were additionally available to ensure that, for telehealth sessions, patients or caregivers had access to needed technology, that their equipment was working, and that the patient or caregiver had the knowledge, skill, and confidence to engage with their providers via telehealth. Our model in the PSCRC is that CHWs are employed by the CBOs, rather than the healthcare institutions, but that shared workplans or Memoranda of Understanding are executed so that the CHWs can work closely with the sickle cell teams.

### 2.3 Survey and Study Procedures

A series of 30 questions were asked in the survey, beginning with site of care; clinical characteristics (SCD type); and type of telehealth visit (phone, video, or both). The telehealth survey included items drawn from the *COVID‐19 Coll*e*ction* from the *consensus measures of Phenotype and Exposure* toolkit, (*PhenX* [17]), with the goal of allowing study results to be compared with other populations across the United States. These 14 items were rated on a 4‐point Likert scale (*strongly disagree*, *disagree*, *agree*, *strongly agree*) along with “don't know” and “not applicable.” The survey covered issues with technology (quality of the connection for seeing/hearing during the call); comfort with telehealth versus in‐person visits; consequences of telehealth visit (availability of an in‐person alternative for that visit and potential impact on healthcare); ability to communicate via telehealth; and enthusiasm for telehealth (level of overall satisfaction, willingness to schedule another telehealth visit or recommend doing so to others). Participants provided information on their sociodemographics: age, gender identity; head of household education; and insurance. Adults with SCD (18 years and older) answered the questions for themselves while parents/caregivers of patients under the age of 18 responded for their child. Survey participants were also able to provide open‐ended responses about their experiences with telehealth at the end of the survey.

All data were de‐identified and stored in a Research Electronic Data Capture (REDCap) database, a web‐based application designed to support data capture for research studies in compliance with HIPAA regulations [18]. REDCap features secure web authentication, data logging, and Secure Sockets Layer (SSL) encryption.

### 2.4 Data Analysis

For this descriptive study, baseline characteristics and distributions of variables are presented as frequencies and percentages for categorical variables, as well as means and standard deviations (SDs) for normally distributed continuous variables and medians and interquartile ranges (IQRs) for non‐normally distributed continuous variables. Categorical variables were analyzed using *χ* ^2^, or Fisher's exact test for sparse tables. Continuous variables were compared using *t* test or Mann–Whitney *U* test, as appropriate. We further organized variables into domains from the literature on telehealth including: experience with telehealth (previous participation, timing of most recent visit, Wi‐Fi access, support at home, expressed interest in navigator); experience with systems for telehealth (trouble with hearing or seeing provider and vice versa, unstable Internet connection, did not have needed device, concerns with privacy, uncomfortable being on video, instructions hard to follow); understandability of health information shared and ability to ask questions; and consumer focus (telehealth visit started on time, provider was able to address health concerns, comfort level with telehealth, would not have been able to see provider without telehealth, felt healthcare was better because of telehealth). The domain of satisfaction included overall satisfaction, would recommend telehealth to friends and family, willing to take part in telehealth again, and prefer telehealth for routine SCD care.

A multivariable logistic regression model was used to evaluate the relations between independent variables and our outcome of overall satisfaction with telehealth (“top‐box” or highest rating). Our model was adjusted for gender identity and patient status (adult or pediatric) as potential confounders of the relations between the independent variables and the outcome. Other variables were selected based on significant associations (two‐tailed *p* < 0.05) in univariate analyses from each telehealth domain. Statistical analyses were conducted using Stata v17.0 (StataCorp LLC, College Station, TX).

We qualitatively analyzed comments that participants entered as free text using content and thematic analysis [19]. One coder (Y.L.) reviewed the responses, grouped them into initial categories, and created a codebook of the categories. A second coder (M.G.) independently reviewed the responses using the codebook and created additional categories for the responses as needed. The two coders came together to resolve any discrepancies by consensus, with input from the senior author (M.T.). Following this process, we determined that the data sufficiently captured the iteratively derived themes, with little new variation.

## 3 Results

While 332 respondents consented to the study, 85 indicated that they/their child had not participated in telehealth and two did not complete the survey for unknown reasons. Therefore, the sample size was *n* = 245 participants in the analysis including 151 adults 18 years and older with SCD [median (IQR) = 36 (28, 43) years], and 94 parents/caregivers of children with SCD [median child age (IQR) = 12 (7, 14) years; Table 1]. Over half of both adults (62.3%) and children (61.7%) with SCD were identified as female. The most common educational attainment of the head of household was some college (adults—43.7%; parents/caregivers—48.9%). Adults (63.6%) and children (56.4%) were primarily publicly insured (Medicaid and state insurance), with no patients reporting that they were uninsured. About 94% of both adults and parents/caregivers of children reported English as their primary language, with 6 adults (4.0%) and 1 parent/caregiver (1.1%) reporting Spanish as their primary language. Other languages included French, Yoruba, Arabic, Tagalog, and Luo. Most participants (55.1%) resided in California, followed by Nevada and Washington (12.2% each), Arizona (8.2%), Oregon (7.3%), and Colorado (4.9%).

**Table 1:** Participant Sociodemographics (*N* = 245).

Category	*n* (%)	
	Adults with SCD (18 years and older) (*n* = 151)	Children with SCD^a^ (younger than 18 years) (*n* = 94)
Age		
Median (IQR)	36 (28, 43) years	12 (7, 14) years
Gender identity		
Female	94 (62.3)	58 (61.7)
Male	57 (37.7)	36 (38.3)
Highest education—Head of household		
High school graduate or less	25 (16.6)	15 (16.0)
Some college	66 (43.7)	46 (48.9)
Bachelor's and beyond	56 (37.1)	33 (35.1)
Health insurance^b^		
Private	46 (30.5)	37 (39.4)
Public (e.g., Medicare/Medicaid)	96 (63.6)	53 (56.4)
Other	8 (5.3)	4 (4.3)
Primary language		
English	142 (94.0)	88 (93.6)
Spanish	6 (4.0)	1 (1.1)
Other	1 (0.7)	5 (5.3)
State		
Arizona	8 (5.3)	12 (12.8)
California	93 (61.6)	42 (44.7)
Colorado	12 (7.9)	0 (0)
Nevada	12 (7.9)	18 (19.1)
Oregon	11 (7.3)	7 (7.4)
Washington	15 (9.9)	15 (16.0)

^a^ Reported by parents/caregivers.

^b^ Respondents could report more than one insurance. Public insurance includes other government‐sponsored insurance, such as state insurance. Other insurance includes emergency assistance and unknown.

### 3.1 Clinical Characteristics

The majority (60.8%) of patients were diagnosed with sickle cell anemia (SCD‐SS or SCD‐Sβ° thalassemia) per patient or parent/caregiver report, followed by SCD‐SC (26.1%) and Sβ+ thalassemia and other variants (10.6%; Table 2). Most adults and children with SCD were on hydroxyurea by report (57.6%), while more adults (26.5%) than children (14.9%, *p* = 0.03) were on other disease‐modifying therapies. More adults (17.9%) compared with children (6.4%, *p* = 0.01) also reported they were on chronic transfusion therapy. Of adults and children on hydroxyurea (*n* = 141), the majority (67.4%) reported they took the medicine every day.

**Table 2:** Clinical characteristics (*N* = 245).

Category	*n* (%)	
	Adults with SCD (18 years and older)	Children with SCD^a^ (younger than 18 years)
Sickle cell type		
SS or Sβ^0^ thalassemia	95 (62.9)	53 (56.4)
SC	33 (21.9)	31 (33.0)
Sβ^+^thalassemia and other diagnoses	18 (11.9)	8 (8.5)
Don't know	5 (3.3)	2 (2.1)
Disease‐modifying therapies		
Hydroxyurea	89 (58.9)	52 (55.3)
Others (Crizanlizumab, voxelotor, l‐glutamine)*	40 (26.5)	14 (14.9)
Chronic transfusion therapy*	27 (17.9)	6 (6.4)
Hydroxyurea adherence		
Completely adherent (every day)	54 (60.7)	41 (78.8)
Partially adherent (most days)	24 (27.0)	6 (11.5)
Not adherent (few to no days)	6 (6.7)	2 (3.8)

^a^ Reported by parents/caregivers.

* *p* < 0.05.

### 3.2 Experiences With Telehealth

In Table 3, survey items are grouped into domains and compared for adults and parents/caregivers of children. Within the first domain Experiences with Telehealth, there was a significant difference in relation to timing of the most recent telehealth visit with adults more often reporting their most recent visit was in the last month (46.4%), while more parents/caregivers of children reported the most recent visit was in the last 1–6 months (62.8%, *p* < 0.001). Internet/Wi‐Fi access at home was very high (98% overall) and did not vary by patient age. Parents/caregivers were more likely to report that they had assistance in the home with accessing telehealth (80.9%) compared with adults with SCD (63.6%, *p* = 0.003). Twenty‐three percent of all participants expressed interest in assistance from a telehealth navigator, with no difference between adults with SCD and parents/caregivers.

**Table 3:** Telehealth experience domains and items (*N* = 245).

Domain/items	*n* (%)	
	Adults with SCD (18 years and older)	Children with SCD^a^ (younger than 18 years)
Experience with telehealth		
Most recent telehealth visit***		
In the last month	70 (46.4)	17 (18.1)
1–6 months ago	66 (43.7)	59 (62.8)
More than 6 months ago	15 (9.9)	18 (19.1)
Have Internet and/or Wi‐Fi access	146 (96.7)	94 (100)
Have assistance in the home for accessing telehealth**	96 (63.6)	76 (80.9)
Interest in assistance with telehealth from navigator	31 (20.5)	26 (27.7)
System experience		
Device(s) used during telehealth sessions		
Computer**	56 (37.1)	53 (56.4)
Phone	114 (75.5)	63 (67.0)
Tablet	27 (17.9)	21 (22.3)
Other	2 (1.3)	0 (0)
Issues during telehealth sessions		
Trouble hearing or seeing	12 (4.0)	4 (2.2)
No Internet access	0 (0)	1 (1.1)
Did not have needed technology/device	0 (0)	1 (1.1)
Concerns about privacy	0 (0)	0 (0)
Uncomfortable with video	0 (0)	0 (0)
Instructions hard to follow	0 (0)	2 (2.1)
Information sharing		
Understood what provider was saying	67 (44.3)	52 (55.3)
Provider understood what I was saying	67 (44.3)	49 (52.1)
I could easily ask questions*	70 (46.4)	57 (60.6)
Consumer focus		
Telehealth visit started on time*	56 (37.8)	50 (53.8)
Provider was able to address health concerns	74 (49.7)	56 (60.2)
Felt comfortable with telehealth	112 (83.6)	65 (73.0)
Would not have been able to be seen without telehealth	19 (14.1)	18 (21.2)
Better healthcare as a result of telehealth**	13 (11.6)	21 (27.3)
Overall satisfaction		
Satisfied overall with telehealth*	66 (44.9)	57 (60.6)
Willing to continue to take part in telehealth	67 (45.6)	53 (57.0)
Would recommend telehealth to others*	59 (40.7)	51 (55.4)

^a^ Reported by parents/caregivers.

* *p* < 0.05

** *p* < 0.01

*** *p* < 0.001.

Within the System Experience domain, we found that phones were utilized for most sessions (72.2%). However, parents/caregivers (56.4%) were more likely than adults (37.1%, *p* = 0.003) to use a computer for sessions. Few respondents reported issues with the telehealth sessions, and these were primarily trouble hearing or seeing (6.5% overall), with no differences based on adults versus parents/caregivers reporting. In the information‐sharing domain, about half of all participants indicated that they understood what the provider was saying and vice versa. There was a significant difference between adults and parents/caregivers in response to “I could easily ask questions” with parents/caregivers more likely to respond in the affirmative (60.6% vs. 46.4%, *p* = 0.03).

In the Consumer Focus domain, parents/caregivers reported more often than adults that their visits started on time (53.8% vs. 37.8%, *p* = 0.015) and that they felt they received better healthcare because of taking part in telehealth (27.3% vs. 11.6%, *p* = 0.006). Over half of both groups indicated that their providers were able to address their health concerns (53.1% overall) and the majority expressed that they felt comfortable with telehealth (72.2% overall). Fifteen percent overall indicated they would not have been seen without telehealth with no difference between adults and parents/caregivers.

Parents/caregivers more often gave the highest rating of overall satisfaction with telehealth (60.6%) compared with adults (44.9%, *p* = 0.02) as well as reporting they would recommend telehealth to others (55.4% vs. 40.7%, *p* = 0.03). While not a statistically significant difference, 57% of parents/caregivers compared with 45.6% of adults also indicated they would be willing to continue to take part in telehealth. Adults with SCD and parents/caregivers also did not differ in preference for the regular conduct of sickle cell visits, with the majority (59.2%) expressing a preference for a mix of in‐person and telehealth visits; 22.4% each preferring either in‐person or telehealth only and 2% expressing no preference.

Based on results from univariate models, health insurance status, size of the PSCRC clinical site, how the telehealth visit was conducted (phone, video, or both), and interest in assistance from a telehealth navigator were entered into a logistic regression model for the outcome top‐box for satisfaction with telehealth (yes/no) that controlled for age and gender identity. It can be seen in Table 4 that individuals who needed assistance from a telehealth navigator were 63% less likely to report the highest level of satisfaction than those who did not need help. Patients seen in large centers were more than twice as likely to report the highest level of satisfaction with telehealth compared with those in medium and small centers.

**Table 4:** Logistic regression analysis of associations with top‐box overall satisfaction scores (*N* = 245).

Characteristic/question	Multivariable analysis		
	OR	(95% CI)	*P*‐value
Patient seen			
Adult	Ref		
Child	1.68	(0.94, 2.99)	0.08
Gender identity			
Male	Ref		
Female	1.50	(0.81, 2.78)	0.19
Health insurance^a^			
Private	Ref		
Public (e.g., Medicare/Medicaid)	1.52	(0.84, 2.76)	0.16
Other	0.50	(0.13, 1.82)	0.29
Size of site			
Small (< 100)	Ref		
Medium (100–200)	2.07	(0.99, 4.31)	0.05
Large (> 200)	2.33	(1.21, 4.48)	0.01
How was the telehealth visit conducted?			
Video only	Ref		
Phone only	0.72	(0.32, 1.58)	0.41
Phone and video	1.12	(0.60, 2.08)	0.72
Would you like assistance from a telehealth navigator?			
Yes	0.37	(0.19, 0.71)	0.003

^a^ Respondents could report more than one insurance. Public insurance includes other government‐sponsored insurance, such as state insurance.

Participants provided more than 200 free text responses about what they liked best, liked least, and wanted to see improved with telehealth. The overwhelming majority commented positively about the efficiency of telehealth and, consistent with survey results, reported very few problems with accessibility.

(I) don't have to stop what I am doing (household tasks), to get there, don't have to be concerned with finding transportation or a babysitter. Don't have to worry about COVID exposure or being around anyone sick or anything.

(Adult with SCD)

Didn't have to travel with my child who was in pain.

(Parent/caregiver of child with SCD)

The importance of not having to worry about childcare was cited by more adults compared with parents/caregivers, whereas more parents/caregivers reported improved pain care for their child as a benefit of telehealth. Areas of needed improvement cited by participants included provider‐related factors (e.g., starting the visit on time; having less background noise in the provider's office) and integration of multidisciplinary services, such as mental health.

There were two providers in the office conducting telehealth visits, so it was difficult to decipher what was being said to us.

(Parent/caregiver of child with SCD)

Be on time. The clinic reprimands the patient for being late, but there is no consequence or accountability when the clinic is late.

(Adult with SCD)

My telehealth care would be better if it would integrate my psychological needs and providers with my healthcare team.

(Adult with SCD)

More adults commented on missing the face‐to‐face interaction with their provider.

(I don't like having to) describe pain without being seen in person.

(Adult with SCD)

The doctors can't perform a physical.

(Adult with SCD)

Nevertheless, expanding telehealth and maintaining accessibility were top priorities for all participants.

(Telehealth) needs to continue even when COVID restrictions lift.

(Parent/caregiver of child with SCD)

## 4 Discussion

In this survey study of adults with SCD and parents/caregivers of children in a large geographic region in the Western United States, we found almost all our survey participants (~90%) reported average to high overall satisfaction with telehealth. Our results are similar to overall satisfaction ratings (~86%) found across a range of other populations, settings, and conditions [20]. Both caregivers and adults with SCD were positive regarding information sharing between themselves and their providers, comfort level with telehealth, and how well their child's or their health concerns were addressed. However, caregivers were generally more positive about their telehealth experiences, more often giving the highest overall rating and noting they could easily ask questions of their providers, felt they received better care via telehealth versus in‐person, and even reporting more often that their visits started on time.

We found similarities and differences in our results compared with those from recent studies of caregivers' experiences of telehealth for their children with SCD in the Midwest [21, 22]. Caregivers participating in the studies in the Midwest and in our study in the West cited the importance of telehealth for removing barriers of distance and transportation and they expressed appreciation for their dedicated teams of SCD specialists. Caregivers who had participated with the direct‐to‐consumer model of telehealth in the Midwest indicated that a disadvantage was the lack of a hands‐on physical examination. In the present study, adults with SCD, not caregivers, voiced skepticism about assessment and pain care without face‐to‐face interaction. The caregivers in the Midwest highlighted concerns about provider knowledge and bias contributing to inequities in SCD care. While our survey did not solicit comprehensive information about barriers to care, adolescents and adults in other studies conducted in our region certainly highlight provider lack of knowledge and implicit bias as seriously impacting care received and willingness to seek care [23]. Across the US healthcare system, individuals with SCD continue to face considerable difficulties with access to and receipt of high‐quality healthcare due to structural and interpersonal racism, so mistrust is well founded. It will be crucial to ensure that telehealth does not contribute to inequities based on age, location (e.g., rural, urban), or setting (e.g., academic, community). Research is needed to understand interactions among such factors as manifestations of structural racism varying by geographic location that might lend to distinct experiences.

In the present study, adults with SCD were more likely to say they had been seen in the last month compared with pediatric patients. It is not clear if they were in fact seen more frequently, but their reports are consistent with other literature that telehealth makes it easier for patients/families to attend appointments without having to leave home/work or defer other activities to come to the clinic to be seen [24]. Results from a recent retrospective study of over 474,000 electronic health records covering the years 2020–2022, showed there were fewer “no shows” with telehealth as compared to in‐person clinic visits with improvements in no‐show rates greatest for Indigenous and Black/African American patients [25]. Our PSCRC clinicians noted that post the height of the pandemic, patients were given the option of telehealth or in‐person visits and if the patient did need to be seen more frequently (e.g., in the case of filling and monitoring prescriptions for opioids) telehealth made this more feasible. Our clinicians also indicated they felt comfortable with the quality of the virtual assessments and do not express feeling any greater burden as the use of telehealth remains steady.

The majority of both adults and parents/caregivers of children with SCD expressed a preference for a mix of in‐person and telehealth, rather than one modality versus the other, similar to findings from other studies with SCD populations [8, 26]. Just as individualized care planning for SCD is associated with improved outcomes in the acute setting, it may be appropriate for providers to routinely assess the ideal combination of telehealth and in‐person visits based on patient/family needs and preferences, as well as delivery of high quality, comprehensive care [27, 28, 29]. Research is needed on patient‐reported and other health outcomes in SCD in relation to the conduct of in‐person or telehealth visits for different types of concerns and acute and chronic complications. As previously noted, any impact of telehealth utilization on provider–patient interactions, rapport, trust, and communication should be carefully monitored. A holistic approach to assessing burdens associated with SCD conditions on individuals and families should include not only such factors as lost time from school and work and other caregiving responsibilities in the home [21], but how telehealth either might add to or relieve these burdens.

While few accessibility or technical issues were cited by participants in the current study, parents/caregivers of children reported more often than adults with SCD that they had support in the home for telehealth. The parents/caregivers reported better access to computers, which may have improved the quality of the visits and it is possible that this difference in how telehealth visits were accessed may have contributed to a less‐than‐optimal sense of ability to communicate and receive the best care on the part of adults [4, 30]. In the year between the first PSCRC survey and the current study, participation in telehealth almost doubled, reliable Internet access remained high, and support at home increased for parents/caregivers of children with SCD [15]. Interest in support from a telehealth navigator was less with the present study compared with previously, and overall satisfaction was greater for those who did not express interest in such assistance. It is encouraging that our population with SCD, who typically grapples with many negative social drivers of health, maintained or gained the ability to utilize technology for their routine healthcare [31]. However, few participants in the present or earlier PSCRC survey reported a primary language other than English, so there remains a need to understand the experiences of telehealth for these individuals. Hispanic/LatinX populations have been found to face more barriers with telehealth compared with Whites and non‐Hispanic Blacks in the United States, so will be an important population to include in future research [32]. Ongoing evaluation of the deployment of telehealth in SCD must ensure that it does not contribute to any widening of the digital divide based on age, language, geographic location, or other social factors [33].

Limitations of the current study include that we were not able to utilize a true pre–post‐survey methodology, so potentially very different people may have participated in the present study compared with the initial PSCRC survey. Notably, participation in telehealth doubled in the interval between the first PSCRC survey, so our current sample may represent a different population, thus limiting generalizability of findings. The PSCRC survey population encompassed western states and primarily publicly insured patients, so the findings may not be applicable to other regions of the United States nor to uninsured patients. We could not make assumptions that parents/caregivers of children and adults with SCD were always seen in pediatric versus adult centers. “Adults” might still be in pediatric centers given the variability in time of transfer and we were not able to distinguish if patients were seen in lifespan centers. Finally, this study relied on self‐reported data which is susceptible to participant and recall bias.

## 5 Conclusions

Our study highlights the favorability of telehealth, the limited issues overall experienced using telehealth—both from a technology and consumer focus—and the positive impact on SCD care among adults and children with SCD in the Western region of the United States during the COVID‐19 pandemic. This study revealed parents/caregivers of children, and adults with SCD, reported higher levels of telehealth satisfaction in large centers. This suggests a need to support smaller and medium‐sized centers that may have been less facile in implementation, or that may serve rural or underserved populations that have been found to face the most challenges with accessing healthcare. This study also draws attention to the need for future studies to understand the experiences of telehealth for a broader population, especially those who speak languages other than English. Most participants preferred a mix of in‐person and telehealth visits, suggesting the need for ongoing policies to support access to telehealth to maximize SCD care, well beyond the pandemic. For example, payers, healthcare institutions, and policy makers could ensure that proper video equipment is available to all. Funds or waivers could be provided to give affordable, strong, and reliable Internet access to those living with SCD, as well as other chronic conditions.

Telehealth shows promise to reduce healthcare inequities as populations with SCD have the opportunity to receive the healthcare they need and deserve, regardless of social or economic status, or geographic location. However, we must remain vigilant that differences in digital literacy or access to technology do not, in fact, lead to increased disparities in access to comprehensive SCD care across all regions of the United States.

## Author Contributions

**Marsha Treadwell:** conceptualization; investigation; funding acquisition; writing–original draft; methodology; validation; visualization; writing–review and editing; formal analysis; project administration; supervision; resources; data curation. **Lisa Du:** data curation; writing–review and editing; investigation; methodology; validation; formal analysis. **Yemi Lawrence:** investigation; writing–review and editing; methodology; data curation; validation; formal analysis. **Maxine Gaspar:** investigation; methodology; validation; writing–review and editing; data curation; formal analysis. **Kathryn Hassell:** conceptualization; investigation; funding acquisition; writing–review and editing; methodology. **Sanjay Shah:** conceptualization; investigation; funding acquisition; writing–review and editing; methodology. **Moses Akpan:** investigation; writing–review and editing. **Nicole Crook:** investigation; writing–review and editing; validation. **Marcia Taylor:** investigation; writing–review and editing. **Srila Gopal:** conceptualization; investigation; funding acquisition; methodology; writing–review and editing. All authors have read and approved the final version of the manuscript.

## Ethics Statement

University of California, San Francisco Institutional Review Board Approval # 20‐31178.

## Consent

Participants followed a link to the survey and upon reviewing a study summary and risks/benefits, they provided electronic consent for their participation.

## Conflicts of Interest

The authors declare no conflicts of interest.

## Transparency Statement

The lead author Marsha Treadwell affirms that this manuscript is an honest, accurate, and transparent account of the study being reported; that no important aspects of the study have been omitted; and that any discrepancies from the study as planned (and, if relevant, registered) have been explained.

## Supporting information

Supporting information.

## Data Availability

The data supporting this study's findings are available from the corresponding author upon reasonable request. Marsha Treadwell, PhD had full access to all the data in this study and takes complete responsibility for the integrity of the data and the accuracy of the data analysis.
