# Supplementary material for: Tracking Telehealth Needs for Individuals With Sickle Cell Disease Through the COVID‐19 Pandemic: A Cross‐Sectional Survey Study
Source: Health Sci Rep. 2024 Nov 5;7(11):e70163. doi: 10.1002/hsr2.70163 (PMC11538030; doi:10.1002/hsr2.70163)
Supplement: Supplementary file 1 — Supporting information. [file HSR2-7-e70163-s001.pdf]

Telehealth Patient Survey

CONSENT FORM

Introduction & Study Summary

We are asking you to consider taking part in a research study about using telehealth for sickle cell disease care. This study is conducted by Marsha Treadwell from UCSF Benioff Children's Hospital Oakland. Its purpose is to better understand if telehealth can be used to effectively meet with patients with sickle cell disease in the future.

You are being asked to participate because you (or your child) have sickle cell disease.

If you choose to participate and consent to this study, you will be asked to complete a 5 to 10-minute survey about your (or your child's) recent telehealth visit. The survey will ask you to think about your satisfaction with the visit, what went well, and what could have gone better. It will also ask you for demographic information like your sickle cell diagnosis and your age. Data from this study may help us a build a case for why telehealth should be reimbursed for patients with sickle cell disease by insurance carriers.

Being in this study is optional. If you do not wish to consent and participate in this study, you can choose "No, I do NOT consent to being this study" at the bottom of this page. You are free to skip any questions that you prefer not to answer or exit the survey at any time to stop being in the study. You won't be penalized or lose any benefits for which you otherwise qualify. If you choose to participate, any data that was collected for the study will remain as part of the study records and cannot be removed.

Risks & Benefits

There are no significant risks to you if you decide to participate. No identifying information about you will be collected on the survey – in other words, the survey is anonymous. We will keep the information you provide confidential by assigning a unique ID code number to your survey responses. There will be no document that links the ID code number to your name. The main risks to you if you participate are accidental loss of information, but we will do everything possible to avoid any loss of data and make sure that the personal information gathered for this study is kept private.

We do not expect this study to benefit you directly. However, we hope that others may benefit in the future from what we learn as a result of this study.

Costs & Compensation

You will not have any costs for being in this research study. You will not be paid for being in this research study.

Contact Information for Questions

If you have questions about this study in the future, you can contact Dr. Marsha Treadwell at (510) 428-3356. If you have questions or concerns about your rights as a research participant, you can call the UCSF Institutional Review Board at (415) 476-1814.

PARTICIPATION IN RESEARCH IS VOLUNTARY. You have the right to decline to be in this study, or to withdraw from it at any point without penalty or loss of benefits to which you are otherwise entitled.

Do you wish to participate and consent to being in this study?

◯ Yes, I consent to being in this study
◯ No, I do NOT consent to being in this study

TELEHEALTH PATIENT SURVEY

What is Telehealth?

Telehealth is the use of information and communication technologies to provide care when you and your healthcare provider are not in the same place at the same time. With a phone or device with internet access, you can have access to medical care or services through telehealth, including health care visits and counseling.

Date: ________________________________

Have you ever heard of telehealth prior to this survey?

◯ Yes
◯ No

Have you or your child ever participated in telehealth?

◯ Yes
◯ No

If 'No', why didn't you participate in telehealth?

(check all that apply)

☐ I did not have internet access
☐ I did not have the needed technology/device, for example a smartphone or computer
☐ The instructions were too hard to follow
☐ I was concerned about privacy
☐ I was uncomfortable with video
☐ The doctor did not suggest a video visit
☐ None of the above

--- ABOUT THE PERSON WITH SICKLE CELL DISEASE AND THE TELEHEALTH VISIT ---

Who was the person at the recent sickle cell disease telehealth visit?

(please select the patient type)

◯ You
◯ Your child

Was your telehealth visit conducted by telephone or by video?

◯ Phone only
◯ Video only
◯ By phone and video

Which healthcare system did you have your telehealth visit through?

(please select your provider from the drop down list)

◯ ALASKA - Alaska Pediatric Oncology, Anchorage
◯ ARIZONA - Arizona Board of Regents/University of Arizona
◯ ARIZONA - Phoenix Children's Hospital, Phoenix
◯ CALIFORNIA - UCSF Benioff Children's Hospital & Research Center at Oakland, Oakland
◯ CALIFORNIA - Center for Inherited Blood Disorders, Orange
◯ CALIFORNIA - Valley Children's Hospital, Fresno
◯ COLORADO - University of Colorado, Denver
◯ MONTANA - Kalispell Regional Healthcare, Kalispell
◯ NEVADA - Hemostasis and Thrombosis Center of Nevada, Las Vegas
◯ NEW MEXICO - The Regents of the University of New Mexico, Albuquerque
◯ OREGON - Oregon Health & Science University, Portland
◯ UTAH - Intermountain Health Services, Salt Lake City
◯ WASHINGTON STATE - Seattle Children's Hospital, Seattle
◯ NOT LISTED HERE

If you had a sickle cell telehealth visit through another site not listed above, please specify: ________________________________

(please type your answer in the space provided)

--- ABOUT YOUR RECENT TELEHEALTH VISIT ---

When was your most recent telehealth visit?

◯ In the last month
◯ 1-3 months ago
◯ 3-6 months ago
◯ 6-12 months ago
◯ More than a year ago

Do you have access to Internet and/or WIFI at home or on your phone?

◯ Yes
◯ No

What device(s) do you use for your telehealth sessions?

(check all that apply)

☐ Computer
☐ Phone
☐ Tablet
☐ Other: ____________

If 'Other' device, please tell us which:

(type your answer in the space provided) ______________________________

Did you have any issues during your telehealth session?

◯ Yes
◯ No

If 'Yes', what were the issues?

(check all that apply)

☐ I did not have internet access
☐ I did not have the needed technology/device, for example a smartphone or computer
☐ The instructions were too hard to follow
☐ I was concerned about privacy
☐ I was uncomfortable with video
☐ Other: ___________

If 'Other' issue, please briefly describe:

(type your answer in the space provided) ______________________________

Are you familiar with Zoom Meetings and able to download the free app?

◯ Yes
◯ No

Do you have someone at home that may be able to assist you in accessing your telehealth sessions?

◯ Yes
◯ No

If available, would you be interested in having a Telehealth Navigator - Community Health Worker assist you in getting started and provide support with telehealth?

◯ Yes
◯ No

--- ABOUT YOUR RECENT TELEHEALTH VISIT ---Instructions: Please rate the following statements about your recent telehealth visit.

	Strongly disagree	Disagree	Agree	Strongly agree	Don't know	Not applicable
The telehealth visit started on time	◯	◯	◯	◯	◯	◯
I had trouble hearing or seeing the provider	◯	◯	◯	◯	◯	◯
The provider had trouble hearing or seeing me	◯	◯	◯	◯	◯	◯
The provider was able to address my health concerns	◯	◯	◯	◯	◯	◯
I felt comfortable with my healthcare visit being through the telehealth system	◯	◯	◯	◯	◯	◯
I would rather have gone to the regular clinic in person if I could have	◯	◯	◯	◯	◯	◯
I would not have been able to see a provider at all without taking part in the telehealth	◯	◯	◯	◯	◯	◯
I feel I will have better health care results by taking part in telehealth	◯	◯	◯	◯	◯	◯
I understood what the provider was saying	◯	◯	◯	◯	◯	◯
The provider could understand what I was saying	◯	◯	◯	◯	◯	◯
I could easily ask questions	◯	◯	◯	◯	◯	◯
Overall, I am satisfied with my telehealth visit	◯	◯	◯	◯	◯	◯
I am willing to take part in another telehealth visit	◯	◯	◯	◯	◯	◯
I would recommend telehealth at this clinic to others	◯	◯	◯	◯	◯	◯

How do you prefer to have your regular (non-sick) visits with your sickle cell disease provider?

◯ In-person office visit only
◯ By phone telehealth only
◯ By video telehealth only
◯ A mix of telehealth (phone and video)
◯ A mix of in-person and telehealth (phone or video)
◯ No preference

What did you like best about your telehealth visit?

(please type your answer in the space below)

What did you like least about your telehealth visit?

(please type your answer in the space below)

Please tell us how we can make telehealth visits better.

(please type your answer in the space below)

--- ABOUT YOU --- Instructions: Please respond to the following questions.

My sickle cell type is

- ◯ SS
- ◯ SC
- ◯ SB 0 thalassemia
- ◯ SB + thalassemia
- ◯ Other __________
- ◯ Don't know

If 'Other' type, please tell us what type

(please type your answer in the space provided) __________________________________

I am prescribed hydroxyurea medicine for my sickle cell disease

- ◯ Yes
- ◯ No

I remember to take hydroxyurea

- ◯ Every day-almost never miss taking it
- ◯ Most days
- ◯ 1-2 x times a week
- ◯ I don't ever take it

I take the following treatments for my sickle cell disease

(check all that apply)

- ☐ Oxbryta - Voxelotor
- ☐ Adakveo - Crizanlizumab
- ☐ Endari - L-glutamine
- ☐ Regularly scheduled blood transfusions
- ☐ Other __________
- ☐ None

If 'Other' treatment, please tell us which:

(type your answer in the space provided) __________________________________

What is your gender?

- ◯ Male
- ◯ Female
- ◯ Other

How old are you?

(please select your age from the drop down list)

- ◯ < 1 yrs old
- ◯ 1
- ◯ 2
- ◯ 3
- ◯ 4
- ◯ 5
- ◯ 6
- ◯ 7
- ◯ 8
- ◯ 9
- ◯ 10
- ◯ 11
- ◯ 12
- ◯ 13
- ◯ 14
- ◯ 15
- ◯ 16
- ◯ 17
- ◯ 18
- ◯ 19
- ◯ 20
- ◯ 21
- ◯ 22
- ◯ 23
- ◯ 24
- ◯ 25
- ◯ 26
- ◯ 27
- ◯ 28
- ◯ 29
- ◯ 30
- ◯ 31
- ◯ 32
- ◯ 33
- ◯ 34
- ◯ 35
- ◯ 36
- ◯ 37
- ◯ 38
- ◯ 39
- ◯ 40
- ◯ 41
- ◯ 42
- ◯ 43
- ◯ 44
- ◯ 45
- ◯ 46
- ◯ 47
- ◯ 48
- ◯ 49
- ◯ 50
- ◯ 51
- ◯ 52
- ◯ 53
- ◯ 54
- ◯ 55
- ◯ 56
- ◯ 57
- ◯ 58
- ◯ 59
- ◯ 60
- ◯ 61
- ◯ 62
- ◯ 63
- ◯ 64
- ◯ 65
- ◯ 66
- ◯ 67
- ◯ 68

	◯ 69<br>◯ 70<br>◯ 71<br>◯ 72<br>◯ 73<br>◯ 74<br>◯ 75+
What is the highest level of education that the head of your household has completed?<br><br>(please use the drop down list to select the head of your household's highlest level of education)	◯ Never attended/Kindergarten only<br>◯ 1st Grade<br>◯ 2nd Grade<br>◯ 3rd Grade<br>◯ 4th Grade<br>◯ 5th Grade<br>◯ 6th Grade<br>◯ 7th Grade<br>◯ 8th Grade<br>◯ 9th Grade<br>◯ 10th Grade<br>◯ 11th Grade<br>◯ 12th Grade, no diploma<br>◯ High school graduate<br>◯ GED or equivalent<br>◯ Some college, no degree<br>◯ Associate degree: occupational/technical/vocational program<br>◯ Associate degree: academic program<br>◯ Bachelor's degree (e.g., BA, AB, BS, BBA)<br>◯ Master's degree (e.g., MA, MS, MEng, MEd, MBA)<br>◯ Professional school degree (e.g., MD, DDS, DVM, JD)<br>◯ Doctoral degree (e.g., PhD, EdD)<br>◯ Unknown
What type of health insurance do you have?<br><br>◯ I do not have health insurance<br>◯ Private / employer-sponsored insurance<br>◯ Affordable Care / ObamaCare / Marketplace<br>◯ Medicaid<br>◯ Medicare<br>◯ Other ________	
If 'Other' insurance, please tell us what type?<br><br>(type your answer in the space provided)	______________________________
What is your primary language?<br><br>◯ English<br>◯ Spanish<br>◯ Other: ___________	
If 'Other' primary language, please tell us which:<br><br>(type your answer in the space provided)	______________________________
If 'Other' primary language, what changes would help you better use telehealth? Please briefly explain:<br><br>(type your answer in the space provided)	______________________________

What State do you live in?

(please select your state from the drop down list)

- ◯ Alabama
- ◯ Alaska
- ◯ Arizona
- ◯ Arkansas
- ◯ California
- ◯ Colorado
- ◯ Connecticut
- ◯ Delaware
- ◯ District of Columbia
- ◯ Florida
- ◯ Georgia
- ◯ Hawaii
- ◯ Idaho
- ◯ Illinois
- ◯ Indiana
- ◯ Iowa
- ◯ Kansas
- ◯ Kentucky
- ◯ Louisiana
- ◯ Maine
- ◯ Maryland
- ◯ Massachusetts
- ◯ Michigan
- ◯ Minnesota
- ◯ Mississippi
- ◯ Missouri
- ◯ Montana
- ◯ Nebraska
- ◯ Nevada
- ◯ New Hampshire
- ◯ New Jersey
- ◯ New Mexico
- ◯ New York
- ◯ North Carolina
- ◯ North Dakota
- ◯ Ohio
- ◯ Oklahoma
- ◯ Oregon
- ◯ Pennsylvania
- ◯ Puerto Rico
- ◯ Rhode Island
- ◯ South Carolina
- ◯ South Dakota
- ◯ Tennessee
- ◯ Texas
- ◯ Utah
- ◯ Vermont
- ◯ Virginia
- ◯ US Virgin Islands
- ◯ Washington
- ◯ West Virginia
- ◯ Wisconsin
- ◯ Wyoming

--- ABOUT YOUR CHILD'S RECENT TELEHEALTH VISIT ---

When was your child's most recent telehealth visit?

◯ In the last month
◯ 1-3 months ago
◯ 3-6 months ago
◯ 6-12 months ago
◯ More than a year ago

Do you have access to Internet and/or WIFI at home or on your phone?

◯ Yes
◯ No

What device(s) do you use for your child's telehealth sessions?

(check all that apply)

☐ Computer
☐ Phone
☐ Tablet
☐ Other: ___________

If 'Other' device, please tell us which: ________________________________

(type your answer in the space provided)

Did you have any issues during your telehealth session?

◯ Yes
◯ No

If 'Yes', what were the issues?

(check all that apply)

☐ I did not have internet access
☐ I did not have the needed technology/device, for example a smartphone or computer
☐ The instructions were too hard to follow
☐ I was concerned about privacy
☐ I was uncomfortable with video
☐ Other: ____________

If 'Other' issue, please briefly describe: ________________________________

(type your answer in the space provided)

Are you familiar with Zoom Meetings and able to download the free app?

◯ Yes
◯ No

Do you have someone at home that may be able to assist you and your child in accessing your telehealth sessions?

◯ Yes
◯ No

If available, would you be interested in having a Telehealth Navigator - Community Health Worker assist you and your child in getting started and provide support with telehealth?

◯ Yes
◯ No

--- ABOUT YOUR CHILD'S RECENT TELEHEALTH VISIT ---Please rate the following statements about your child's recent telehealth visit.

	Strongly disagree	Disagree	Agree	Strongly agree	Don't know	Not applicable
The telehealth visit started on time	◯	◯	◯	◯	◯	◯
I had trouble hearing or seeing the provider	◯	◯	◯	◯	◯	◯
The provider had trouble hearing or seeing me	◯	◯	◯	◯	◯	◯
The provider was able to address my child's health concerns	◯	◯	◯	◯	◯	◯
I felt comfortable with my child's healthcare visit being through the telehealth system	◯	◯	◯	◯	◯	◯
I would rather have gone to the regular clinic in person if I could have	◯	◯	◯	◯	◯	◯
I would not have been able to see a provider for my child at all without taking part in the telehealth	◯	◯	◯	◯	◯	◯
I feel my child will have better health care results by taking part in telehealth	◯	◯	◯	◯	◯	◯
I understood what the provider was saying	◯	◯	◯	◯	◯	◯
The provider could understand what I was saying	◯	◯	◯	◯	◯	◯
I could easily ask questions	◯	◯	◯	◯	◯	◯
Overall, I am satisfied with my child's telehealth visit	◯	◯	◯	◯	◯	◯
I am willing to take part in another telehealth visit for my child	◯	◯	◯	◯	◯	◯
I would recommend telehealth at this clinic to others	◯	◯	◯	◯	◯	◯

How do you prefer to have your child's regular (non-sick) visits with your sickle cell disease provider?

◯ In-person office visit only
◯ By phone telehealth only
◯ By video telehealth only
◯ A mix of telehealth (phone and video)
◯ A mix of in-person and telehealth (phone or video)
◯ No preference

What did you like best about your child's telehealth visit?

(please type your answer in the space below)

What did you like least about your child's telehealth visit?

(please type your answer in the space below)

Please tell us how we can make telehealth visits better.

(please type your answer in the space below)

--- ABOUT YOUR CHILD ---Instructions: Please respond to the following questions about your child.

Your child's sickle cell type is

◯ SS
◯ SC
◯ SB 0 thalassemia
◯ SB + thalassemia
◯ Other __________
◯ Don't know

If 'Other' type, please tell us what type

(please type your answer in the space provided) __________________________________

Your child is prescribed hydroxyurea medicine for their sickle cell disease

◯ Yes
◯ No

Your child remembers to take hydroxyurea

◯ Every day-almost never miss taking it
◯ Most days
◯ 1-2 x times a week
◯ My child does not ever take it

Your child takes one of the following treatments for their sickle cell disease

(check all that apply)

☐ Oxbryta - Voxelotor
☐ Adakveo - Crizanlizumab
☐ Endari - L-glutamine
☐ Regularly scheduled blood transfusions
☐ Other __________
☐ None

If 'Other' treatment, please tell us which:

(type your answer in the space provided) __________________________________

What is your child's gender?

◯ Male
◯ Female
◯ Other

How old is your chlid?

(please select your child's age from the drop down list)

◯ < 1 yrs old
◯ 1
◯ 2
◯ 3
◯ 4
◯ 5
◯ 6
◯ 7
◯ 8
◯ 9
◯ 10
◯ 11
◯ 12
◯ 13
◯ 14
◯ 15
◯ 16
◯ 17
◯ 18
◯ 19
◯ 20
◯ 21
◯ 22
◯ 23
◯ 24
◯ 25
◯ 26

What is the highest level of school that your child has completed?

(please use the drop down list to select your child's highest level of schooling)

◯ Never attended/Kindergarten only
◯ 1st Grade
◯ 2nd Grade
◯ 3rd Grade
◯ 4th Grade
◯ 5th Grade
◯ 6th Grade
◯ 7th Grade
◯ 8th Grade
◯ 9th Grade
◯ 10th Grade
◯ 11th Grade
◯ 12th Grade, no diploma
◯ High school graduate
◯ GED or equivalent
◯ Some college, no degree
◯ Associate degree: occupational/technical/vocational program
◯ Associate degree: academic program
◯ Bachelor's degree (e.g., BA, AB, BS, BBA)

What type of health insurance does your child have?

◯ I do not have health insurance for my child
◯ Private / employer-sponsored insurance
◯ Affordable Care / ObamaCare / Marketplace
◯ Medicaid
◯ Medicare
◯ Other ________

If 'Other' insurance, please tell us what type?

(type your answer in the space provided)

____

What State does your child live in?

(please select your child's state from the drop down list)

- ◯ Alabama
- ◯ Alaska
- ◯ Arizona
- ◯ Arkansas
- ◯ California
- ◯ Colorado
- ◯ Connecticut
- ◯ Delaware
- ◯ District of Columbia
- ◯ Florida
- ◯ Georgia
- ◯ Hawaii
- ◯ Idaho
- ◯ Illinois
- ◯ Indiana
- ◯ Iowa
- ◯ Kansas
- ◯ Kentucky
- ◯ Louisiana
- ◯ Maine
- ◯ Maryland
- ◯ Massachusetts
- ◯ Michigan
- ◯ Minnesota
- ◯ Mississippi
- ◯ Missouri
- ◯ Montana
- ◯ Nebraska
- ◯ Nevada
- ◯ New Hampshire
- ◯ New Jersey
- ◯ New Mexico
- ◯ New York
- ◯ North Carolina
- ◯ North Dakota
- ◯ Ohio
- ◯ Oklahoma
- ◯ Oregon
- ◯ Pennsylvania
- ◯ Puerto Rico
- ◯ Rhode Island
- ◯ South Carolina
- ◯ South Dakota
- ◯ Tennessee
- ◯ Texas
- ◯ Utah
- ◯ Vermont
- ◯ Virginia
- ◯ US Virgin Islands
- ◯ Washington
- ◯ West Virginia
- ◯ Wisconsin
- ◯ Wyoming

--- ABOUT YOU --- Instructions: Please respond to the following questions.

What is your gender?

- ◯ Male
- ◯ Female
- ◯ Other

How old are you?

(please select your age from the drop down list)

◯ < 1 yrs old
◯ 1
◯ 2
◯ 3
◯ 4
◯ 5
◯ 6
◯ 7
◯ 8
◯ 9
◯ 10
◯ 11
◯ 12
◯ 13
◯ 14
◯ 15
◯ 16
◯ 17
◯ 18
◯ 19
◯ 20
◯ 21
◯ 22
◯ 23
◯ 24
◯ 25
◯ 26
◯ 27
◯ 28
◯ 29
◯ 30
◯ 31
◯ 32
◯ 33
◯ 34
◯ 35
◯ 36
◯ 37
◯ 38
◯ 39
◯ 40
◯ 41
◯ 42
◯ 43
◯ 44
◯ 45
◯ 46
◯ 47
◯ 48
◯ 49
◯ 50
◯ 51
◯ 52
◯ 53
◯ 54
◯ 55
◯ 56
◯ 57
◯ 58
◯ 59
◯ 60
◯ 61
◯ 62
◯ 63
◯ 64
◯ 65
◯ 66
◯ 67
◯ 68

	◯ 69<br>◯ 70<br>◯ 71<br>◯ 72<br>◯ 73<br>◯ 74<br>◯ 75+
What is the highest level of education that the head of your household has completed?<br><br>(please use the drop down list to select the head of your household's highest level of education)	◯ Never attended/Kindergarten only<br>◯ 1st Grade<br>◯ 2nd Grade<br>◯ 3rd Grade<br>◯ 4th Grade<br>◯ 5th Grade<br>◯ 6th Grade<br>◯ 7th Grade<br>◯ 8th Grade<br>◯ 9th Grade<br>◯ 10th Grade<br>◯ 11th Grade<br>◯ 12th Grade, no diploma<br>◯ High school graduate<br>◯ GED or equivalent<br>◯ Some college, no degree<br>◯ Associate degree: occupational/technical/vocational program<br>◯ Associate degree: academic program<br>◯ Bachelor's degree (e.g., BA, AB, BS, BBA)<br>◯ Master's degree (e.g., MA, MS, MEng, MEd, MBA)<br>◯ Professional school degree (e.g., MD, DDS, DVM, JD)<br>◯ Doctoral degree (e.g., PhD, EdD)<br>◯ Unknown
What is your primary language?<br><br>◯ English<br>◯ Spanish<br>◯ Other: ____________	
If 'Other' primary language, please tell us which:<br><br>(type your answer in the space provided)	________________________________
If 'Other' primary language, what changes would help you better use telehealth? Please briefly explain:<br><br>(type your answer in the space provided)	________________________________
Thank you for answering the survey. Please click 'Submit' to finish.	
